# Japanese version of the 42-item psychological well-being scale (PWBS-42): a validation study

**DOI:** 10.1186/s40359-020-00441-1

**Published:** 2020-07-20

**Authors:** Natsu Sasaki, Kazuhiro Watanabe, Kotaro Imamura, Daisuke Nishi, Mayumi Karasawa, Chiemi Kan, Carol Diane Ryff, Norito Kawakami

**Affiliations:** 1 Department of Mental Health, Graduate School of Medicine, The University of Tokyo, 7-3-1, Hongo, Bunkyo-ku, Tokyo, 113-0033 Japan; 2 Department of Communication, Tokyo Women’s Christian University, Tokyo, Japan; 3 National Institute of Occupational Safety and Health, Kiyose, Japan; 4 Department of Psychology/Institute on Aging, University of Wisconsin-Madison, Madison, USA

**Keywords:** Psychological well-being, PWB, Eudaimonic well-being, Positive mental health, Happiness, Meaning in life, Psychometric properties, Reliability, Validity, Flourishing structural invariance

## Abstract

**Background:**

The aim of this study was to examine the internal consistency, structural validity, and convergent/known-group validity of the Japanese version of the 42-item Psychological Well-Being Scale (PWBS-42).

**Methods:**

The PWBS-42 includes six 7-item subscales designed to measure the following dimensions of *eudaimonic* psychological well-being: 1) autonomy, 2) environmental mastery, 3) personal growth, 4) positive relations with others, 5) purpose in life, and 6) self-acceptance. A questionnaire was administered to 2102 community residents in Tokyo aged 30 or over as a part of the Midlife in Japan (MIDJA) survey, in 2008. The internal consistency reliability was tested using Cronbach’s α. Structural validity was examined using exploratory factor analysis (EFA). Convergent validity was evaluated by calculating correlations of the Japanese PWBS-42 subscales with life satisfaction, negative affect, negative adjectives, positive affect, positive adjectives, self-esteem, and perceived stress scales.

**Results:**

Data from 1027 respondents (505 males and 522 females) were analyzed (valid response rate = 56.2%). Cronbach’s α values ranged from 0.70 to 0.78 for five of the subscales, while that for purpose in life was lower (0.57). EFA yielded a five-factor structure: The first two factors consisted of negative and positive items mostly from the environmental mastery, purpose in life, and self-acceptance subscales. The third, fourth, and fifth factors consisted mostly of items from the positive relations with others, autonomy, and personal growth subscales, respectively. As hypothesized, the scores for life satisfaction, negative and positive affect/adjectives, self-esteem and perceived stress were significantly correlated with all subscales of the Japanese PWBS-42.

**Conclusion:**

The subscales of the Japanese version of the PWBS-42 showed accep. levels of reliability and support for convergent validity in the Japanese population. The factor structure was slightly different from the theoretical 6-factor model: items of three subscales (environmental mastery, purpose in life, and self-acceptance) loaded together on two factors. This finding may be interpreted in light of the interdependent self construal found in Japan in which these three components could be closely linked.

## Background

*Eudaimonic* well-being has been often discussed as purpose in life, meaning of life [1, 2], or as a concept relevant to self-determination theory [3, 4]. One of the most famous models of *eudaimonic* well-being is the psychological well-being (PWB) model, developed by Carol D. Ryff [5]. PWB integrated psychological concepts such as self-actualization [6] and being a fully functioning person [7], distilling six key factors of well-being: autonomy, environmental mastery, personal growth, positive relations with others, purpose in life, and self-acceptance. The association of Ryff’s PWB with various health outcomes has been well-documented [8]: overall longer survival [9], low risk of physical diseases such as stroke [10], myocardial infarction [11], and metabolic syndrome [12], and low risk of mental illness such as depression [13, 14], insomnia [15] and Alzheimer’s disease [16]. In terms of biological functioning, higher levels of PWB associated with lower level of plasma cytokines [17, 18].

Since promoting PWB can contribute to people’s personal growth, career development, and successful aging, measuring and promoting PWB among the Japanese population might be helpful for solving important issues in life at every stage. PWB has been frequently measured with Ryff’s *Psychological Well-Being Scales (PWBS)* [19]. The reliability and validity of the PWBS has been established in more than 30 languages and across various cultures [20, 21].

In Japan, three published articles have examined measurement of PWB in Japanese samples [22–24]. However, the measures the researchers used made it difficult to compare scores with those in other languages: two of them were not developed based on Ryff’s original items of PWBS [22, 23], the other one [24] had 84 items which were rated dichotomously, with categories of “Yes” or “No”. However, in cross-cultural cohort studies, it is more common for items to be rated on 6- or 7-point Likert scales. In addition, the measures used in these three studies have limitations in terms of balance between the burden on the participants of completing them and adequate depth of measurement, thus raising questions about the credibility of their assessments of the six well-being constructs. Although the length of the PWBS ranges from 18 [25] to 120 items [19], the ultra-short (18 items) version had psychometric problems of low internal consistency (0.33 < Cronbach’s alpha < 0.56) [25], while acceptable criteria is regarded Cronbach’s alpha > 0.7. Moreover, the 84- and 120-items versions imposed an undue burden on the respondents [8, 19, 26, 27]. The 42-item PWBS has been recently recognized as achieving the needed balance, and has been adopted in several studies including the Midlife in the United States (MIDUS) survey, a large cohort study in the United States [8, 28].

Hence, the purpose of this study is to examine the internal consistency, structural validity, and convergent/known-group validity of the Japanese version of the 42-item PWBS among the general population in Japan. In addition, we considered the interpretability of this measurement.

## Methods

### Study design and participants

This is a validation study for a self-report measure of well-being. The Midlife in Japan (MIDJA) study surveyed 2102 community residents aged 30–79 years old living in the 23 wards of Tokyo city from April to September 2008 [29]. The responses were collected from 1027 adults (505 men and 522 women; age range = 30 to 79 years). The overall response rate was 56.2%. It was found that 275 participants were non-eligible (i.e., undelivered) due to having moved, unknown address, being absent during the time of the survey, illness, injury, being hospitalized or being deceased, which described in detail elsewhere (data available upon request). The primary objective of the MIDJA is to compare the Japanese sample (MIDJA) with the United States sample (MIDUS) to test hypotheses linking constructs of interdependence and independence to well-being and health. All respondents completed a self-administered questionnaire. The Research Ethics Committee of the Graduate School of Medicine in The University of Tokyo approved this study (no. 1691- [5]).

The study was reported according to the Consensus-based Standards for the Selection of Health Measurement Instruments (COSMIN) guideline, which is used to improve the quality of efforts to develop health-related self-report measurement instruments [30]. Each finding for properties of measurement in this study was assessed by the COSMIN Risk of Bias checklist (Additional file 1).

### Measurements

#### Psychological well-being scale (PWBS)

With permission from Professor Ryff, the developer of the PWBS, we translated the 42 items of the PWBS into Japanese. The PWBS [19] originally comprised six subscales with seven items each to measure the six factors: 1) autonomy (e.g., I am not afraid to voice my opinions, even when they are in opposition to the opinions of most people.); 2) environmental mastery (e.g., In general, I feel I am in charge of the situation in which I live.); 3) personal growth(e.g., I think it is important to have new experiences that challenge how I think about myself and the world.); 4) positive relations with others(e.g., Most people see me as loving and affectionate.); 5) purpose in life(e.g., I have a sense of direction and purpose in life.); and 6) self-acceptance(e.g., In general, I feel confident and positive about myself.). Response categories for these items are on a seven-point scale: ranging from 1 (“Strongly disagree”) to 7 (“Strongly agree”). The scores for six subscales were calculated as averages; higher scores mean greater psychological well-being.

The authors complied with the International Society for Pharmacoeconomics and Outcomes Research (ISPOR) taskforce guidelines [31], which constitute the standard procedure for translation and adaptation of self-reporting scales in other languages. The forward (from English to Japanese) translation of the PWBS 42 items was prepared by Prof. Karasawa, who is a research member of the MIDJA, with considerable knowledge about the theoretical background of PWB. The forward translation was reviewed and revised by colleagues at The University of Tokyo to confirm its relevance and meaningfulness. After these consultations with researchers, the slightly amended version was back-translated into English by an independent translator who was blind to the original English version and reviewed by research members of MIDJA. The original developer, Prof. Ryff, reviewed this back translation and further corrections were made based on her suggestions.

#### Life satisfaction (6 items)

Life satisfaction was measured by the newly developed six items scale for the MIDUS/MIDJA (e.g., “How would you rate your life overall these days”). Assessing overall, work, health, family (the mean of ratings for relationship with spouse/partner and relationship with children) and financial satisfaction each question asked participants about their satisfaction with their life on current days on a scale ranging from 0 (the worst possible) to 10 (the best possible). The overall score was constructed by calculating the mean of the items. The scale was computed for cases that had valid values for at least one item on the scale. Higher scores reflect higher levels of overall life satisfaction, meaning high *hedonic* well-being. In this study sample, the internal consistency reliability of the scale (Cronbach’s alpha) was 0.75 [32].

#### Positive and negative affect/adjective

Positive and negative adjectives were measured by four and five items of each (e.g., enthusiastic, attentive, proud, active, afraid, jittery, irritable, ashamed, upset) from *The Positive and Negative Affect Schedule (PANAS)* [33], which is a widely used mood measurement and regarded as one of the indicators of *hedonic* well-being. In addition, positive affect was each measured by six items (i.e., cheerful, in good spirits, extremely happy, calm and peaceful, satisfied, full of life), because such low arousal positive feeling has been reported to have stronger link with health among Japanese people, other than high arousal feelings, measured by PANAS [34]. The negative affect was also measured by an original six-item scale consisting one item on “nervousness” from PANAS and items from other sources (i.e., so sad nothing could cheer you up, restless or fidgety, hopeless, that everything was an effort, worthless). Additional items both in positive and negative affect were retrieved from the previous study [35]. This study used the past 30 days as the time frame (i.e.., During the past 30 days, how much of the time did you feel…). All items were rated on a 5-point Likert-type scale, ranging from 1 “None of the time” to 5 “All of the time”. Scales were constructed by calculating the mean across each set of items. The scales were computed for cases that had valid values for at least one item on the scale. In this study sample, the internal consistency reliability (Cronbach’s alpha) of the scales for 1) positive affect, 2) positive adjective, 3) negative affect, 4) negative adjective was 1) 0.93, 2) 0.80, 3) 0.86 and 4) 0.82 respectively [32]. We used these four sub-scales as a measurement of affect to keep their independent construction. Positive affect and adjective in this questionnaire is well validated elsewhere [34].

#### Self-esteem (7 items)

Self-esteem was measured with a scale consisting of seven items taken from Rosenberg’s self-esteem scales [36] (e.g., “I am able to do things as well as most people”). A response to each item was scored on a seven-point scale ranging from 1 (agree strongly) to 7 (disagree strongly). In this study sample, Cronbach’s alpha for men and women was 0.63 and 0.69 [37], respectively.

#### Perceived stress (10 items)

Perceived stress was measured with the Perceived Stress Scale (PSS) [38, 39], which asked 10 items about their recognized stressors on these 30 days (e.g., “felt that you were unable to control the important things in your life?”). Each item was rated on five-point scale ranging from 1 (Never) to 5 (Very often). In this study sample, Cronbach’s alpha was 0.76 [37].

#### Demographic variables

A questionnaire was administered to assess demographic variables, including gender (male or female), age, education status (Junior high school, some high school, high school, vocational school, 2 year college, some college, 4 or 6 year college or over graduate school), marital status (married, divorced/widowed or single) and work status (Have a paid job or Do not have a paid job).

### Statistical analysis

Statistical significance was defined as *p* < 0.05. All the statistical analyses were performed using SPSS 26.0, Japanese version (SPSS Inc., Chicago, IL).

Before conducting the analyses, the scores of some items were reversed as recommended in Ryff’s original PWBS [19]. For an item with a missing value, the mean value of the completed items was imputed. The scales were computed for cases that had valid values for at least one item in each domain following the documentation of “Scales and Constructed Variables in MIDJA” guide [32].

To assess the internal consistency of the Japanese PWBS, Cronbach’s alpha (α) coefficients were calculated for each of the six subscales. To assess structural validity, confirmatory factor analysis (CFA) was conducted to test the goodness of fit for the existing structure of psychological well-being, and an exploratory factor analysis (EFA) was conducted in case the CFA showed a poor fit. Based on previous research, we hypothesized a six-factor structure. In addition, we extracted factors with eigenvalues of more than 1.0, following the Kaiser–Guttman “Eigenvalues greater than one” criterion [40–42], using robust maximum likelihood estimation. In the two EFAs, the promax rotation method was adopted to calculate factor loadings for the items. As a hypothesis test for convergent validity, Pearson’s correlation coefficients (rs) were calculated between each score of the PWBS and life satisfaction, positive and negative affect/adjectives, and perceived stress, which was considered to have moderate-to-strong associations with psychological well-being. Positive and moderate correlations would be expected with life satisfaction, positive affect/adjectives, and self-esteem. Negative and moderate correlations would be expected with negative affect/adjectives and perceived stress. Although these measures consisted of distinct components of well-being, prior research revealed relations among *evaluative*, *hedonic* and *eudaimonic* well-being [43]. In addition, a one-way factorial ANOVA was performed as another hypothesis test for known-group validity to compare the scores of the six subscales of the Japanese PWBS when stratified based on demographic and occupational variables (sex, age, marital status and working status). For these demographic and occupational variables, we observed significant differences from the PWB scores in previous studies (e.g., high personal growth and purpose in life in the elderly, high positive relationship with others in women) [25].

## Results

### Characteristics of participants

We received responses from 1027 members (56.2%) of the target sample. The proportion of males was 49.2%, and average age was 54.4 years old. The demographic characteristics of the participants are shown in Table 1. Many of the participants were married (69.1%) and had a paid job (71.6%).

**Table 1:** Participant characteristics. (*N* = 1027)

	N (%)
Sex(male)	505 (49.2)
Age mean, SD	54.4, 14.1
30–39	200 (19.5)
40–49	215 (20.9)
50–59	206 (20.1)
60–69	200 (19.5)
70≦	206 (20.1)
Marital status	
Married	710 (69.1)
Divorsed/Widowed	149 (14.5)
Single	166 (16.2)
Missing	2 (0.2)
Working status	
Have a paid job	735 (71.6)
No paid job	289 (28.1)
Missing	3 (0.3)
Education	
Junior high school	97 (9.4)
Some high school	33 (3.2)
High school	306 (29.8)
Vocational school	139 (13.5)
2 year college	89 (8.7)
Some college	26 (2.5)
4 or 6 year college	300 (29.2)
Over graduate school	25 (2.4)
Missing	12 (1.2)

### Internal consistency

Table 2 shows mean scores and Cronbach’s alphas (α) for the PWBS-42 subscales. The Cronbach’s α coefficients ranged from 0.70 to 0.78, except for that for Purpose in life (α = 0.57).

**Table 2:** Average, standard deviation (SD) and reliability among Japanese population for the PWB domains. (N = 1027)

PWB ^a^ domains (Possible range: 7–49)	Mean	SD	Cronbach’s α
Autonomy	30.6	5.3	0.70
Environmental Mastery	31.7	5.4	0.74
Personal growth	33.8	5.6	0.75
Positive relationship with others	33.5	5.7	0.76
Purpose in life	31.8	5.0	0.57
Self-acceptance	30.8	5.7	0.78

Participants who had missing data were analyzed according to the algorithm described in the Method section

*SD* standard deviation

^a^*PWB* Psychological well-being

### Factor structure of PWBS-42 in Japanese

The results of CFA are shown in Table 3. The original hypothesized six-factor model demonstrated poor fit (χ^2^ [804] = 6651, CFI = 0.638, TLI = 0.594, RMSEA = 0.084). The results of EFA assuming a six factor structure indicated that the 42 items loaded on five of the six factors and no item loaded on the sixth factor (data available upon request**)**. Then, we tried conducting another EFA that hypothesized a 5-factor structure with the promax rotation method, which allows factors to correlate with each other, using a robust maximum likelihood estimation. Table 4 shows that the EFA then successfully yielded five factors. The first two factors consisted of negative and positive items mostly from environmental mastery, purpose in life, and self-acceptance. The third, fourth, and fifth factors consisted mostly of items for positive relations with others, autonomy, and personal growth, respectively. EFA according to the Kaiser–Guttman’s criterion indicated an eight-factor structure (data available upon request). Authors did not adopt the model because it was difficult to interpret the meaningfulness of the eight factors and there were few items loaded on the subordinate factors.

**Table 3:** Factor loadings of PWB 42 items and model fit in confirmatory factor analyses

Model fit			6-factor		
χ2(df)			6651(804)		
CFI			0.638		
TLI			0.594		
RMSEA(95%CI)			0.084(0.082–0.086)		
**Factor loadings**^a^					
Autonomy		Environmental mastery		Personal growth	
A1	0.676	E1	0.595	G1	0.434
A2	0.448	E2	0.429	G2	0.317
A3	0.402	E3	0.485	G3	0.675
A4	0.402	E4	0.526	G4	0.630
A5	0.660	E5	0.496	G5	0.565
A6	0.529	E6	0.667	G6	0.695
A7	0.351	E7	0.552	G7	0.382
Positive relation with others		Purpose in life		Self-acceptance	
R1	0.477	P1	0.233	S1	0.554
R2	0.531	P2	0.654	S2	0.666
R3	0.660	P3	0.664	S3	0.341
R4	0.563	P4	0.509	S4	0.615
R5	0.405	P5	0.635	S5	0.674
R6	0.681	P6	0.414	S6	0.578
R7	0.382	P7	−0.230	S7	0.639

^a^ All items showed significant factor loading

**Table 4:** Exploratory factor analysis assuming a five-factor structure by using maximum likelihood with Promax rotation

	Reverse	Item	Factor loading score				
			1	2	3	4	5
E6	R	Difficult arranging life in satisfying way	**0.682**	0.170	−0.019	0.059	− 0.090
E2	R	Demands of everyday life often get me down	**0.633**	0.031	−0.101	0.008	−0.107
E5	R	Overwhelmed by my responsibilities	**0.628**	0.023	−0.134	0.135	−0.065
A6	R	Worry about what others think of me	**0.600**	0.070	−0.147	0.236	−0.175
S5	R	Disappointed about achievements in life	**0.592**	0.165	0.101	−0.118	0.058
P4	R	Daily activities seem trivial & unimportant	**0.531**	0.057	−0.015	− 0.089	0.153
R2	R	Maintaining close relationships difficult	**0.531**	−0.109	0.212	0.073	−0.043
S6	R	Self attitude not as positive as others	**0.520**	0.222	0–.029	−0.127	0.048
S3	R	Others have gotten more out of life than me	**0.507**	−0.032	−0.082	0.018	0.001
R3	R	Few close friends to share concerns with	**0.501**	−0.076	0.339	−0.088	0.037
E3	R	Don’t fit in with people and community	**0.492**	−0.074	0.264	−0.045	− 0.012
G3	R	Haven’t improved as person over years	**0.488**	0.285	−0.013	−0.140	0.143
R6	R	No experience with warm & trusting relations	**0.487**	−0.135	0.300	−0.094	0.231
G6	R	Gave up trying to make improvements long ago	**0.461**	0.065	0.025	−0.036	0.397
P3	R	No good sense of what I’m trying to accomplish	**0.458**	0.401	−0.160	−0.139	0.150
G7	R	Do not enjoy situations requiring a change in my ways	**0.441**	−0.129	−0.005	0.097	0.135
A3	R	Influenced by people with strong opinions	**0.432**	−0.158	−0.115	0.305	−0.079
S7		Feel good when compare myself to friends	0.071	**0.703**	0.174	−0.021	−0.184
P7	R	Done all there is to do in life	0.059	**−0.684**	0.067	0.047	0.350
S2		Feel positive/confident about self	0.081	**0.595**	−0.085	0.209	0.146
G4		Developed a lot as person over time	0.052	**0.560**	0.183	−0.003	0.036
P5		Enjoy making plans for future and making it real	−0.091	**0.520**	−.044	−0.002	0.379
P2		Have sense of direction/purpose in life	−0.100	**0.509**	−0.142	0.115	0.483
S1		Pleased with how life turned out	0.103	**0.480**	0.196	−0.082	−0.057
E4		Good at managing daily responsibilities	0.051	**0.465**	0.131	0.241	−0.113
A4		Confidence in my opinions even if others are contrary	−0.221	**0.427**	−0.018	0.371	0.083
S4		Like most aspects of my personality	0.147	**0.424**	0.283	0.075	−0.136
E7		Able to build lifestyle to my liking	0.148	**0.424**	0.176	0.100	0–.105
P6		Some wander aimlessly but not me	−0.040	**0.369**	0.113	−0.011	0.082
R7		I can trust friends & they can trust me	0.003	0.144	**0.679**	0.073	−0.092
R4		Enjoy conversations with family & friends	−0.004	0.132	**0.508**	0.037	0.080
R1		Most see me as loving/affectionate	−0.094	0.191	**0.495**	0.051	0.000
R5		Others describe me as giving/share time	−0.116	0.292	**0.386**	0.019	−0.025
A1		Not afraid to voice opposing opinions	0.095	− 0.020	0.037	**0.694**	0.103
A5	R	Difficulty voicing opinion on controversial issues	0.461	−0.147	− 0.068	**0.498**	0.138
E1		In charge of situation in which I live	0.110	0.142	0.207	**0.450**	0.070
A2		Decisions not influenced by others’ actions	−0.019	0.286	0–.007	**0.389**	−0.088
A7		Judge self by what I think is important	−0.090	0.070	0.102	**0.379**	0.038
G5		Life process of learning/changing/growth	−0.133	0.217	0.214	0.047	**0.460**
G2		Important to experience the challenge how to think about yourself and the world	−0.233	− 0.037	0.199	0.236	**0.447**
G1	R	Not interested in activities to expand horizons	0.296	−0.231	0.043	0.087	**0.446**
P1	R	Live life day to day, don’t think about future	0.202	−0.132	−0.185	− 0.075	**0.432**

Table 5 shows correlations between the scores of the theoretical subscales that originally assumed on the PWBS-42 and scores of the five factors extracted from the previous EFA. Each factor score was sum of items. There were significant and exclusive correlation between the five factors and the six subscales of the PWBS-42; Factor 2 and Self-acceptance, Factor 3 and Positive relationship with others, Factor 4 and Autonomy, and Factor 5 and Personal growth. Factor 1 showed moderate association with all theoretical six subscales.

**Table 5:** Pearson’s correlation coefficients^a^ between the each score of PWB and each score of factors which extracted from factorial analysis

Variables [number of item] (minimum-maximum)	N	Mean	SD	Psychological well-being (PWB) domains					
				Autonomy	Environmental Mastery	Personal growth	Positive relationship with others	Purpose in life	Self-acceptance
Factor 1 [17] (33–119)	1024	77.65	14.0	0.508**	0.825**	0.734**	0.718**	0.629**	0.724**
Factor 2 [12] (17–78)	1021	53.49	8.4	0.499**	0.638**	0.646**	0.572**	0.667**	0.811**
Factor 3 [4] (4–28)	1025	18.77	3.3	0.256**	0.456**	0.498**	0.853**	0.402**	0.524**
Factor 4 [5] (10–35)	1021	22.67	4.1	0.896**	0.556**	0.446**	0.357**	0.339**	0.442**
Factor 5 [4] (8–28)	1024	19.65	3.3	0.267**	0.338**	0.771**	0.444**	0.646**	0.349**

*SD* standard deviation

^a^ Participants who had missing data were excluded from each analysis

** *p* < 0.01, * < 0.05

### Hypothesis testing

1. **Convergent validity**

Table 6 shows correlations between the scores of the six subscales of the PWBS-42 and life satisfaction, positive and negative affect/adjectives, self-esteem, and perceived stress. Although the EFA showed a five-factor structure, we presented average scores and correlations of the original six subscales because it was planned as part of validation and for keeping the international comparability. In addition, we cannot conclude that the original six-factor structure and the observed five-factor structure are definitely different as shown in Table 5. All scales were significantly correlated with all domains of PWB. Scales related to negative feelings (i.e., negative affect, adjective, PSS) were correlated negatively with all domains (− 0.55 < r < − 0.13). Positive scales (i.e., life satisfaction, positive affect, adjectives, self-esteem) were correlated positively with all domains (0.24 < r < 0.71).

2. **Known-group validity**

**Table 6:** Pearson’s correlation coefficients^a^ between each subscale on the PWB and Life Satisfaction, Negative Affect, Negative Adjectives, Positive Affect, Positive Adjectives, Self-esteem, Percieved Stress Scale (PSS) among Japanese population

Variables (possible range)	N	Mean	SD	Psychological well-being (PWB) domains					
				Autonomy	Environmental Mastery	Personal growth	Positive relationship with others	Purpose in life	Self-acceptance
Life Satisfaction (0–10)	1027	6.10	1.58	0.235**	0.528**	0.382**	0.412**	0.357**	0.562**
Positive and negative affect									
Negative Affect (1–5)	1024	1.69	0.65	−0.212**	−0.419**	−0.218**	−0.293**	−0.207**	−0.408**
Negative Adjectives (1–5)	1021	1.89	0.66	−0.245**	− 0.354**	−0.126**	− 0.205**	−0.147**	− 0.285**
Positive Affect [1–3]	1022	3.24	0.76	0.220**	0.446**	0.380**	0.464**	0.302**	0.509**
Positive Adjectives (1–5)	1020	3.07	0.76	0.344**	0.427**	0.421**	0.394**	0.350**	0.445**
Self-esteem (7–49)	1021	31.02	5.58	0.463**	0.646**	0.509**	0.470**	0.433**	0.714**
PSS (10–50)	1008	26.11	5.77	−0.306**	−0.554**	− 0.298**	−0.370**	− 0.295**	−0.511**

*SD* standard deviation

^a^ Participants who had missing data were excluded from each analysis

** p < 0.01, * < 0.05

Table 7 shows the results of ANOVAs to examine the differences between each category for the demographic characteristics (sex, age, marital and working status). One-way ANOVA revealed some significant differences among variables (e.g., high autonomy in males, high personal growth in workers).

**Table 7:** Comparison with the participant characteristics for four scales of PWBS-42 in Japanese (One-way ANOVA) (N = 1027)

	Autonomy			Environmental Mastery			Personal growth		
Category (N)	Mean (SD)	F	p	Mean (SD)	F	p	Mean (SD)	F	p
Sex		12.86	< 0.001		3.15	0.076		6.52	0.011
Male (505)	31.2(5.3)			31.4(5.8)			33.3(5.6)		
Female (522)	30.1(5.2)			32.0(5.0)			34.2(5.6)		
Age		4.02	0.003		4.03	0.003		6.66	< 0.001
30–39 (200)	29.5(5.9)			30.6(5.3)			34.5(5.9)		
40–49 (215)	30.4(5.7)			31.5(5.4)			34.7(5.5)		
50–59 (206)	30.8(5.4)			32.5(6.0)			34.3(5.8)		
60–69 (200)	31.2(4.9)			32.2(5.5)			33.1(5.6)		
70≦ (206)	31.3(4.4)			31.6(4.5)			32.4(5.0)		
Marital status		0.53	0.586		11.25	< 0.001		8.26	< 0.001
Married (710)	30.7(5.3)			32.2(5.4)			34.2(5.5)		
Divorced/Widowed (149)	30.6(4.7)			31.3(5.3)			33.1(5.8)		
Single (166)	30.2(6.0)			30.0(5.3)			32.4(5.9)		
Working status		1.87	0.171		0.20	0.656		15.39	< 0.001
Have a paid job (735)	30.8(5.5)			31.8(5.6)			34.2(5.8)		
No paid job (289)	30.3(4.9)			31.6(5.0)			32.7(5.1)		
	**Positive relationship with others**			**Purpose in life**			**Self-acceptance**		
Category (N)	Mean (SD)	F	p	Mean (SD)	F	p	Mean (SD)	F	P
Sex		30.12	< 0.001		0.75	0.387		2.87	0.091
Male (505)	32.5(6.0)			31.6(5.2)			30.5(5.7)		
Female (522)	34.5(5.3)			31.9(4.8)			31.1(5.7)		
Age		0.912	0.456		2.66	0.032		1.47	0.209
30–39 (200)	33.4(6.1)			32.2(5.5)			30.3(6.3)		
40–49 (215)	33.5(6.2)			31.9(5.1)			31.1(6.2)		
50–59 (206)	34.1(5.6)			32.4(5.1)			31.5(5.9)		
60–69 (200)	33.5(5.3)			31.3(5.1)			30.4(5.1)		
70≦ (206)	33.0(5.4)			31.1(4.3)			30.7(4.7)		
Marital status		15.56	< 0.001		12.61	< 0.001		21.28	< 0.001
Married (710)	34.1(5.4)			32.2(5.0)			31.5(5.5)		
Divorced/Widowed (149)	32.9(5.5)			31.5(5.0)			29.9(6.2)		
Single(166)	31.5(6.6)			30.1(4.9)			28.6(5.4)		
Working status		0.11	0.745		8.85	0.003		1.06	0.304
Have a paid job (735)	33.6(5.9)			32.1(5.1)			31.0(5.9)		
No paid job (289)	33.4(5.2)			31.0(4.8)			30.5(5.1)		

*SD* standard deviation

## Discussion

The results showed an acceptable level of internal consistency reliability of the newly translated Japanese version of the PWBS-42, although careful consideration of the results was need given that factor analysis showed a five-factor structure. Cronbach’s alpha coefficient was lower for the scales for purpose in life, possibly because one item of “Done all there is to do in life” (the item P7) behaved differently than the other items. The results of CFA did not show a good model fit for the previously proposed six-factor structure model. The correlation analysis showed theoretically high correlation with other scales, supporting convergent validity. The ANOVA confirmed the hypothesized differences according to demographic characteristics between known independent groups (population in U.S. [25] vs. Japan), suggesting good known-group validity, as described in detail below.

We found that the original six-factor model [5] did not fit well with the present data in the CFA. Rather, the EFA suggested a five-factor structure. The first two factors consisted of negatively and positively worded items mostly from environmental mastery, purpose in life, and self-acceptance, as well as some items from the other subscales. The result for the correlation analysis also showed that Factor 1 and 2 was highly related (r > 0.8) with environmental mastery and self-acceptance. Factor 1 had high correlation (r > 0.7) with most of PWB subscales instead of autonomy and purpose in life. The highest correlation among them was with environmental mastery, identifying the EFA result that the top three items of high loading score in Factor 1 were all about environmental mastery. The correlation of Factor 2 and self-acceptance may possibly be due to relatively high loading scores of S7 and S2. Although purpose in life did not show high correlation with any factors, it might be due to its low internal consistency. We discuss two aspects of the possible reasons the factor analysis yielded these results.

First, it seems that respondents in this sample of Japanese community residents perceived three constructs measured by these subscales (i.e., environmental mastery, purpose in life, and self-acceptance) quite similar to each other. The findings contract to previous observations in Western countries that these constructs are separate in factor analyses [25, 44]. Among Eastern cultures, the self is viewed as interdependent, rather than independent as it is in Western culture. Experiencing interdependence entails seeing oneself as part of a network of social relationships and recognizing that one’s behavior is determined by the others’ perspectives or expectations. The fundamental connectedness of the human community with the surrounding context “self-in-relation-to-other” is focal in individual experiences in Eastern cultures [45]. With the interdependent self, well-being is realized in one’s relationship with surrounding realities, both social and non-social [46]. Thus, environmental mastery, being able to take adaptive and situationally bound actions in the social context, might be closely linked with self-acceptance in a country with an Eastern culture, such as Japan. In addition, also in line with the construal of interdependent cultures, the self becomes most meaningful (i.e., high purpose in life) when it is cast in the appropriate social settings [45]. Japanese people are motivated to find a way to fit in with relevant surroundings by fulfilling obligations to become part of various interpersonal relationships [45]. Purpose in life may be also closely related to environmental mastery and self-acceptance in Japanese culture. In Japanese culture, these three components of PWB may come together and be hard to distinguish.

Second, negative and positive items were divided into two different factors in this sample. The same pattern was shown in a previous study with a UK cohort [47]. There are two reasons that the PWB scale includes negative/positive distinction of wordings; (1) assessing constructs with items framed positively and negatively is needed as a check on acquiescence response bias (i.e., the tendency to agree with everything, perhaps due to not even reading the items); (2) items for the PWB scales were generated based on the theoretical definitions of each construct which included a definition of both high and low scorers for each dimension (i.e., items intended to capture both having and not having the various aspects of well-being) [48]. The use of both negatively and positively worded items is expected to increase the validity of what is meant by having a high score on any dimension of well-being because to get a high score, a person has to both agree with positively worded items and disagree with negatively worded items. The pattern that negatively and positively worded items load on different factors in a factor analysis has been observed for several mental health and psychological well-being scales in Japan [49–52], which is interpreted as differential response styles to negatively and positively worded items in the Japanese population. Thus, the finding for the two factors may not necessarily imply that there are two distinct unique constructs of psychological well-being in the Japanese population. The two factors observed in the exploratory factor analysis may be just due to a reporting style, but still reflect the same construct.

Most items for the remaining three subscales (i.e., positive relations with others, autonomy, and personal growth) loaded on separate factors that seem to reflect the corresponding dimensions of the PWB. The results for the correlation analysis also suggested that Factor 3, 4, and 5 had high to moderate correlation with positive relationship with others, autonomy, and personal growth respectively. The observed five factor-structure in this study seem compatible with the original six-factor model [19] to some extent, while the three subscales (i.e, environmental mastery, purpose in life, and self-acceptance) were collapsed to represent a single combined dimension in this sample. It is possible that the factor structure is affected by the characteristics (e.g., gender, age groups, and urban/rural difference) of the population and the cultural context. The authors could not conclude from this study that the five-factor structure fit to the Japanese PWBS-42 rather than the original six-factor model [19]. Further research is needed to re-examine the factor structure of the Japanese PWBS-42 in a sample with a broader age range and from non-urban settings to know if the pattern could be generalized to the whole Japanese population.

The results for the correlation analysis indicated that all the PWBS-42 correlated highly with other psychometrics variables measured by other scales in the theoretically expected direction, supporting its convergent validity. As shown by ANOVA, there were some significant demographic differences in scores on the Japanese version of the PWBS-42. Specifically, some differences, such as decremental age profiles (with scores for the older people significantly lower than those for younger people) for personal growth and purpose in life, sex profile (with women scoring higher than men) of positive relationship with others, were coincident with previous research in the U.S. [25]. Otherwise, high autonomy in men seems peculiar to Japanese men. Marriage had an advantage over being divorced or single in many subscales, except for autonomy. Previous studies suggested that marriage is a powerful predictor of high well-being [53, 54]. Having paid work was related to high scores for personal growth and purpose in life. These two subscales were found to contribute to work and career related outcomes [55]. The findings supported the view that using six subscales for the Japanese population was appropriate, suggesting known-group validity compared with other population. Regarding interpretability of this scale, the result showed relatively low PWB scores among Japanese sample (30.6 < mean < 33.8) compared with the U.S. sample (37.1 < mean < 40.6). If we need to categorize individuals into low, optimal, and excessively high scores for each dimension of the PWB, such as in assessment for well-being therapy [56], this cultural difference must be considered.

### Limitations

This study has some limitations. First, the MIDJA population consisted of people over 30, so young population under 29 cannot be considered. Second, the translation process is not partially appropriate. Third is that our Japanese participants treated item P7 (see Table 4) as positive even though it is negative phrased (and reversed-scored). We kept this item and its original scoring in the Japanese version of the PWBS-42 in this study to maintain consistency with the original PWBS; however, that practice should be reconsidered in future research. Fourth, some scales which were used in convergent/known-class validity analysis are biased due to insufficient consideration of reliability and validity. Finally, some important properties of the scale were not investigated in the study due to the lack of repeated measurement: test-retest reliability, measurement error, predictive validity and responsiveness.

## Conclusion

The Japanese version of the PWBS-42 showed acceptable reliability and convergent validity when applying the original scoring method for the six subscales. However, the factorial validity based on the original six-factor model was not well supported in this study, while most factors observed in this study seem compatible with the original factors. A further examination is needed to know if the factor structure of the PWBS-42 is different for the Japanese population.

## Supplementary information

**Additional file 1.**

## Data Availability

The data supporting the findings of this study are available from N.Sasaki, but restrictions apply to the availability of these data, which were used under license for the current study, and so are not publicly available. Data is available from the authors upon reasonable request and with permission from N.Kawakami.

## Abbreviations

ANOVA: Analysis of variance;
CFA: Confirmatory factor analysis;
CFI: Comparative fit index;
COSMIN: COnsensus-based Standards for the Selection of Health Measurement Instruments;
EFA: Exploratory factor analysis;
MIDJA: Midlife in Japan survey;
MIDUS: Midlife in the United States survey;
PANAS: The Positive and Negative Affect Schedule;
PSS: The Perceived Stress Scale;
PWB: Psychological Well-Being;
PWBS: Psychological Well-Being Scale;
RMSEA: Root mean square error of approximation;
TLI: Tucker-Levis index;
